# Lifetime prevalence of questionable health behaviors and their psychological roots: A preregistered nationally representative survey

**DOI:** 10.1371/journal.pone.0313173

**Published:** 2024-11-06

**Authors:** Goran Knežević, Marija B. Petrović, Milica Ninković, Zorana Zupan, Petar Lukić, Danka Purić, Marko Živanović, Predrag Teovanović, Sanda Stanković, Iris Žeželj

**Affiliations:** 1 Department of Psychology, Faculty of Philosophy, University of Belgrade, Belgrade, Serbia; 2 LIRA Lab, Faculty of Philosophy, University of Belgrade, Belgrade, Serbia; 3 Faculty of Philosophy, Institute of Psychology, University of Belgrade, Belgrade, Serbia; 4 Faculty of Special Education and Rehabilitation, University of Belgrade, Belgrade, Serbia; Makerere University, UGANDA

## Abstract

A growing body of evidence suggests that questionable health behaviors- not following medical recommendations and resorting to non-evidence based treatments—are more frequent than previously thought, and that they seem to have strong psychological roots. We thus aimed to: 1) document the lifetime prevalence of intentional non-adherence to medical recommendations (iNAR) and use of traditional, complementary and alternative medicine (TCAM) in Serbia and 2) understand how they relate to ‘distal’ psychological factors—personality traits and thinking dispositions, and ‘proximal’ factors—a set of beliefs and cognitive biases under the term ‘irrational mindset’. In this preregistered cross-sectional study on a nationally representative sample (N = 1003), we observed high lifetime prevalence of iNAR (91.3%) and TCAM (99.2%). Irrational beliefs, especially magical health beliefs and medical conspiracy theories, were the strongest predictors of TCAM. They also mediated the relation between Disintegration/lower cognitive reflectiveness and TCAM. High Disintegration, and low Conscientiousness predicted iNAR directly, whilst negative experiences with the healthcare system facilitated both types of questionable health practices. The established psychological profile of people prone to questionable health behaviors and the fact they can be tracked to negative experiences with the system can be used to tailor public health communications.

## Introduction

Paradoxically, while individuals may readily use prescription medication without prescription in their pursuit of maintaining or improving health, they may be hesitant to pursue treatments directly recommended by their physician. Similarly, individuals may readily embrace health-related practices that lack scientific support, but scrutinize the officially prescribed dosage of a medicine. Such health practices can be subsumed under the term “questionable health behaviors”, and further categorized into two broad categories: intentional non-adherence to medical recommendations (iNAR; [1, 2]) and the use of traditional, complementary, and alternative medical practices (TCAM). Both iNAR and TCAM use can have severe health consequences and further increase the burden on healthcare systems: non-adherence can increase mortality rates and the spread of infectious diseases [3], whilst the use of TCAM practices can lead to adverse events [4], interact with official treatment [5, 6] or divert individuals from it [7, 8].

Non-adherence, referring to deviations from official medical recommendations, can stem from multiple external factors, such as the quality and accessibility of the healthcare system or socio-economic conditions [9]. However, here we focus specifically on *intentional* non-adherence: a deliberate decision not to follow medical recommendations, which includes behaviors such as self-medication, changing the dosage or duration of the prescribed medication, or skipping regular medical check-ups. Previous studies have shown that iNAR behaviors capture a single underlying tendency [1], and are partially rooted in psychological factors [2, 10].

TCAM encompasses a broad set of practices that often lack an evidence-base and are not typically integrated into the official medical system [11]. Despite their growing use, TCAM practices remain insufficiently assessed, with existing evidence showing little to no support for their effectiveness [4]. Moreover, the lack of systematic monitoring of their adverse effects makes the safety of TCAM practices highly questionable, whilst they are at the same time advertised as a natural (and thus safe) alternative [12]. For example, herbal teas and supplements made from the germander plant are traditionally used as a natural and safe treatment for diabetes, although they have been banned in some countries after being reported to cause severe hepatotoxicity [13]. Despite this, research shows that TCAM behaviors that assume visiting practitioners are quite prevalent, although this prevalence is highly context-dependent. For example, one multinational study showed that around 50% of respondents from China or Philippines have visited TCAM practitioners, while the same stands for around 36% of respondents from France or 17% of respondents in the Netherlands and 6% from Poland [14]. Prevalence of TCAM use in some countries, like Serbia, are still unknown. The Law on Healthcare in Serbia classifies TCAM into recognized traditional, complementary, and alternative methods for diagnosis, treatment, and rehabilitation. These include practices such as acupuncture, homeopathy, Ayurveda, and traditional Chinese medicine, which are accepted by healthcare authorities as potentially beneficial. Providers of these practices must be licensed by the Ministry of Health, following specific educational and certification requirements. However, these services are not covered by health insurance and must be paid for by patients.

Our previous findings suggest that the use of TCAM practices can be classified into four domains: 1) alternative medical systems, such as homeopathy and quantum medicine; 2) New Age practices, such as crystal healing; 3) natural product-based treatments that encompass a wide set of herbal remedies and supplements, and 4) rituals and customs, mostly capturing traditional and religious customs, such as prayer for health, or wearing a red thread [15]. Similar to iNAR, but even more strongly, the use of TCAM practices seem to be rooted in psychological factors.

### Questionable health behaviors, irrational beliefs and related psychological dispositions

Cognitive fallacies that have been consistently linked to questionable health behaviors, and especially TCAM, encompass beliefs, judgments, decisions, and conclusions that depart from normative rationality, and have been labeled under the umbrella term of the *irrational mindset* [10, 15, 16]. Variables comprising the irrational mindset are treated as “proximal” in our study design as they are conceptually closer to questionable health behaviors, and their content sometimes overlaps. Within the irrational mindset, we can discern between different layers of irrational beliefs. One layer includes irrational beliefs that persist despite being unsupported by evidence. This layer includes superstition, extrasensory beliefs, conspiracy mentality, belief in medical conspiracy theories, magical health beliefs, as well as simultaneous endorsement of contradictory beliefs (i.e. doublethink)—all previously shown to predict TCAM use [15, 17–21]. As a novelty, we also included a different type of irrational beliefs that originate from the cognitive-behavioral psychotherapeutic framework are personal irrational beliefs—rigid, extreme, and unrealistic beliefs about oneself and the world [22]. Limited previous findings suggest that these beliefs are related to intentional non-adherence [10]. The second layer of irrational mindset consists of cognitive biases, which are systematically flawed responses to judgment and decision problems, relatively independent of their content. There is scattered evidence of susceptibility to naturalness bias [15, 23], illusory correlations [15], and belief bias [10], being related to TCAM use. The third layer of the irrational mindset is most content-independent and concerns basic perceptive/cognitive processes. It is the tendency to perceive patterns in randomness, i.e., to make sense of the world by identifying meaningful relations between unrelated stimuli [24] which is called apophenia.

Although the results are less robust in comparison to TCAM use, there is some evidence that aspects of the irrational mindset, such as conspiratorial beliefs [20], personal irrational beliefs [10], and overconfidence bias [16, 25] are related to medical non-adherence as well. However, iNAR seems to be predominantly related to more systemic factors, such as mistrust in the healthcare system and healthcare providers and previous negative experiences with them [1, 26]. Additionally, iNAR and the use of pseudoscientific health practices have been related to lower trust in science [23].

When it comes to ideological roots of these health behaviors, some studies reported that a more conservative political orientation predicts both iNAR [27, 28] and TCAM [29]. Religiosity and spirituality were also found to be predictive [30–33], especially for TCAM practices that are closely related to religion and tradition [15].

Finally, questionable health practices can be traced back to more fundamental, basic psychological dispositions, such as basic personality traits and thinking dispositions [34]. We treated these as distal factors in our study as they encompass basic individual difference variables and do not overlap content-wise with questionable health behaviors. The basic personality traits are usually defined within the HEXACO model [35, 36] and can be further complemented with Disintegration—a reconceptualization of the proneness to psychotic-like experiences as a general dispositional tendency that is not adequately represented by any of the most influential contemporary personality models [37–39]. Recent findings suggest that both iNAR and the use of TCAM are related to high Disintegration, low Honesty, and low Conscientiousness [10]. A similar pattern has also emerged during the COVID-19 pandemic—high Disintegration was predictive of both pandemic-specific TCAM behaviors, as well as non-adherence to COVID-19 guidelines, which were also related to low Honesty [34]. Given the established relationship between Disintegration and questionable health behaviors and previous findings showing that Disintegration is the most robust personality correlate of different aspects of the irrational mindset such as apophenia, conspiratorial beliefs, superstitions, and magical health beliefs [10, 40, 41], the relation between Disintegration and TCAM use should be at least partially mediated by different aspects of the irrational mindset.

While personality traits refer to behavioral predispositions, thinking styles refer to predispositions in information processing. They can be best understood via two contrasting tendencies—to rely on reason and process information analytically or to rely on intuition and synthetical processing. They are typically assessed through self-report scales such as rational/experiential thinking styles [42] or actively open-minded thinking [43, 44]. A similar, but performance-based construct, is called ‘cognitive reflection’—an ability to override intuitive, but incorrect answers on simple numerical/logical tasks [45]. Positive attitudes towards TCAM and its use have been consistently linked to a higher experiential and a lower rational thinking style [46–49] and lower cognitive reflection [50]. There is also some evidence that experiential thinking is related to negative attitudes towards evidence-based practices, which can be treated as a proxy for non-adherence [47], while studies relating cognitive reflection and non-adherence yield inconclusive results [16, 51].

If we describe irrational beliefs as a manifestation of a more superficial approach to information processing—one that implies a lack of cognitive reflection, a preference for experiential thinking, as well as less readiness to alter one’s own beliefs, i.e., less actively open-minded thinking [52, 53]—irrational beliefs should at least partially mediate the effect of this superficial information processing style on TCAM.

### The current study

The current study measured the lifetime prevalence of two types of questionable health behaviors, iNAR and TCAM, on a nationally representative probability sample in Serbia. To understand the roots of these behaviors, we explored the predictive power of sociopolitical variables, healthcare-related beliefs and experiences, personality traits and thinking styles (distal psychological factors), and irrational beliefs and biases (proximal psychological factors, layers within irrational mindset), for both iNAR and TCAM. Both distal and proximal factors are used as predictors in the regression analyses. While the relations between socio political variables and health behaviors have been extensively studied [54, 55], accumulated data suggests that psychological variables might be of greater importance, especially for TCAM [1, 10, 15]. Moreover, previous studies have been conducted on community samples, so data from nationally representative studies is lacking.

To map the mechanism of the effect, we tested the partial mediation effect of apophenia, conspiratorial beliefs, superstitiousness, and magical health beliefs between Disintegration and TCAM, as well as between cognitive reflection and TCAM. We opted to focus on TCAM as the outcome of the mediation model, given that the relations of iNAR with psychological predictors found in previous studies [1] were less robust. Finally, the most robust previous findings regarding the role of the aforementioned predictors in questionable health behaviors [1, 10] were further tested in a comprehensive structural equation model (SEM).

### Hypotheses

We preregistered the hypotheses in our protocol paper [56]. In the first hypothesis, we expected that a minimum of 90% of the general population in Serbia will have used at least some TCAM practices and engaged in at least some iNAR behaviors (H1). Next, we predicted that the correlation between TCAM and iNAR in the general population will be positive, and small to medium in magnitude (H2). We then specified that psychological predictors (distal and proximal factors) would have an incremental predictive value over sociodemographic and health-related variables, and that psychological predictors would explain more variance in both TCAM and iNAR than sociodemographic and health-related variables (H3). Finally, we expected that belief in conspiracy theories, superstitiousness, magical health beliefs, and apophenia would mediate the relationships between Disintegration (H4a) and low cognitive reflection (H4b), as distal predictors, and TCAM as an outcome variable. Analyses included testing simple partial mediation effects and/or SEM models.

## Method

Ethical Committees of the Faculty of Philosophy in Belgrade (#935/1), Faculty of Special Education and Rehabilitation (#139/1), and Faculty of Media and Communication (#228) approved the protocol. All participants signed informed consent (it was provided to them in a written form) and voluntarily participated in the study. All procedures were performed in accordance with relevant guidelines/regulations and adhered to the principles of the Declaration of Helsinki. The study protocol was pre-registered (Trial registration number NCT05808660; Open Science Framework (https://osf.io/gfp4q/, Protocol: https://bmjopen.bmj.com/content/bmjopen/13/10/e075274.full.pdf). A large part of the method section was published as part of the protocol [56].

### Sample

As explained in the protocol paper, a sample of *N* = 1043 enabled detecting a correlation of .10 with a power of .90 (with a two-sided alpha level set at .05). Moreover, this sample size enables detecting even small indirect (mediated) effects in path analyses of simple partial mediation models; It is also adequate for SEM analysis of these models, provided that factor loadings are large enough, and the number of latent factor indicators is equal or larger than three, according to a recent simulation analysis [57].

#### Procedure of data collection

Data collection was conducted via a professional research agency from June 15 to July 5, 2023. The recruited study sample (*N* = 1044) was probabilistic at the household level and designed to be representative of the general population in Serbia between 18 and 75 years of age. Data were collected online and face-to-face via Computer Assisted Personal Interviews (CAPI), using a standardized case report form. Approximately 60% of the sample was recruited online (18–54 years), and 40% face-to-face (55–75 years). The online subsample was a one-staged stratified quota sample. The primary sampling unit was the respondent, a member of an online panel database aged 18–50 years who fulfills the criteria based on population quotas. The face-to-face subsample was a three-stage random representative stratified sample with the statistical circle area as the primary sampling unit, the household as the secondary sampling unit, and the respondent as the tertiary sampling unit. Areas were selected with probability proportional to size, households were selected based on simple random sampling without replacement (random choice of the starting point and equal steps of choice), and the respondents were selected randomly from listed members of the household aged 55–75 years. Trained interviewers specialized in large-sample survey data collection conducted the face-to-face interviews.

If a respondent had a visual impairment or had difficulties reading, the interviewer read out the items and recorded the responses. There was no missing data because the questionnaire had a forced response format. Four attention-check items were dispersed in the questionnaire to exclude participants who failed to provide correct responses to any of them (as recommended for this type of study [58–60], thus increasing the quality of collected data without compromising their validity [61]). The inclusion criterion was age, while the initial exclusion criteria were the inability to understand the Serbian language and failure to pass any of the four attention-check questions (187 participants failed to pass attention check items, resulting in 1044 valid protocols).

#### Data cleaning

After the data were collected, we excluded participants who produced low-quality data due to responding carelessly, i.e.who automatically selected the responses without carefully reading the questions [62]. For the two inventories assessing health-related behavior (iNAR-12 and TCAM-22), we considered maximum scores as indicative of careless responding due to low probability that an individual would engage in *all* TCAM practices or disregard *all* health recommendations. This was empirically supported by density plots (S1 Fig). For HEXACO-60, we computed the standard deviation of responses for each participant, identifying potentially invalid entries through intraindividual *SD* values below 0.70, as suggested by Lee and Ashton [63]. In the case of REI, AOT, and ESB (the only remaining inventories that contained both positively and negatively worded items), we considered a standard deviation of zero as an indicator of careless responding (except when the mean of all items from a particular instrument equaled three, which represented the midpoint of the five-point scale). Combining data from all six inventories, we constructed a combined quality check variable with a score range spanning from zero (no detected instances of careless responding) to six (careless responding identified on all assessments). We excluded 41 individuals (82.9% females, *M*_age_ = 36.32, *SD*_age_ = 16.57) with two or more instances of careless responding, leaving us with the final sample of 1003 participants.

#### Sample representativeness

Although designed to be representative of the Serbian population, our sample deviated from the population quota in some aspects, especially regarding education and region. To obtain more precise estimations of population parameters, we applied case weights to correct these deviations. We then compared population quotas on gender, age, education, and regional distribution from the latest Serbian census data provided by the Statistical Office of the Republic of Serbia (https://www.stat.gov.rs) with those observed in the sample. The distributions of socio-demographic variables in a weighted sample mirrored those in the population, with some tolerable departures observed in age and education (S1 Table).

### Instruments and variables

To assess TCAM and iNAR, we used two instruments—*iNAR-12* [1] a 12-item self-report scale, and *TCAM-22* [15], a 22-item self-report scale. The latter includes four TCAM domains (alternative medical systems, natural product-based practices, New Age practices, and rituals/customs). In both instruments, participants used a binary scale (0—No, 1—Yes) to report lifetime engagement in the listed practices. The instruction emphasized that the use needs to be for health reasons, i.e. for prevention or treatment for disease or any use that advances health. To make sure participants followed the instructions, we illustrated non-health and health use in an example (garlic and honey products). Additionally, participants reported their typical way of use of the TCAM practices (preventive, alternative or complementary) for each of the four TCAM domains.

Sociodemographic variables included participants’ gender, age, education, and place of residence (*urban/rural*). Health variables included body mass index (BMI, based on self-reported weight and height), smoking (*smoker/non-smoker*), self-reported health status (1 –*very poor* to 5 –*very good*), and presence of chronic illness (assessed by indicating responses on a checklist of chronic conditions, e.g., cardiovascular, gastrointestinal, oncological).

Distal predictors included *basic personality traits* of the HEXACO model complemented by Disintegration, thinking styles, and cognitive reflection. The HEXACO-60 inventory [64]; Serbian version [65], includes six broad personality traits (Honesty/Humility, Emotionality, eXtraversion, Agreeableness, Conscientiousness, Openness), each assessed with 10 items (response scale ranging from 1 –*fully disagree* to 5 –*fully agree*). *Disintegration* was assessed using a 20-item version of the DELTA scale [38], capturing nine subdimensions of proneness to psychotic-like experiences and behaviors (response scale ranging from 1 –*fully disagree*, to 5 –*fully agree*). *Thinking styles* were assessed using a short, eight-item version [66] of The Rational-Experiential Inventory [67], with rational and experiential scales consisting of four items each (response scale ranging from 1 –*definitely not true for myself*, to 5 –*definitely true for myself*). The eight-item *Actively Open-Minded Thinking Scale* (response scale ranging from 1 –*completely disagree*, to 5 –*completely agree*), was used to assess the tendency to use evidence to revise beliefs [68]. *Cognitive reflection* was assessed using the short cognitive reflection test (CRT) [45], consisting of three items that cue an intuitive but incorrect response.

The variables within the set of proximal predictors can be classified into those capturing the irrational mindset and those assessing socio-political attitudes and beliefs. The variables of irrational mindset included general and medical conspiratory beliefs, magical health beliefs, superstitiousness, belief in extra-sensory perception, proneness to doublethink, personal irrational beliefs, perceptual apophenia, and five cognitive biases–overconfidence bias, illusory correlation, omission bias, naturalness bias, belief bias, and commitment bias.

*Belief in conspiracy theories* was measured using two scales: the general *Conspiracy Mentality Questionnaire* (CMQ [69]; Serbian version [70], five items), and a more specific assessment measure of *Belief in medical conspiracy theories* (adapted [20, 70, 71], five items), both rated using a 5-point scale (ranging from 1 –*completely disagree*, to 5 –*completely agre*e). We also assessed magical health beliefs with 10 items from the general magical beliefs factor of the *Magical Beliefs about Food and Health Scale* [21]. To assess superstitiousness, we used five items from the Superstition scale [72] with the highest loadings on the general factor, while for the assessment of belief in extra-sensory perception, we used six items with the highest loadings from the *Extra-sensory perception belief scale* (ESB) [73] (ranging from 1 –*completely disagree*, to 4 –*completely agre*e). Doublethink was assessed via the *Proneness to doublethink scale* [74], consisting of 11 pairs of contradictory beliefs (ranging from 1 –*completely disagree*, to 4 –*completely agree*), with the score calculated by counting the number of contradictory pairs for which participants indicated both statements to be true (mark 3 or 4 on a 4-point scale). Personal irrational beliefs were assessed via the short six-item *General Attitude and Belief Scale* (GABS [75] (ranging from 1 –*completely disagree*, to 5 –*completely agree*). *Apophenia* was assessed with the Snowy Pictures Task [76]. The task consists of 24 stimuli, each containing a grainy image, with half of the stimuli containing an embedded object that was used as distractors, while the other half, target stimuli, did not contain any object. The number of objects detected in stimuli where there was none served as a score of apophenia. *Overconfidence bias* was expressed as a difference between the CRT confidence score calculated as the mean percentage of confidence judgment across three items and the accuracy score on the same test calculated as the percentage of correct answers. A single-item measure was used to assess *illusory correlation* [77], where participants were presented with data showing no correlation between two variables [78] and asked to describe the data. Responses indicating a positive correlation between the two variables are treated as evidence of an illusory correlation. *Naturalness bias* was measured as a hypothetical preference for a natural drug over a synthetic drug, all other things being equal [79]. *Omission bias* was assessed by a scenario where refusing a medication is associated with a higher risk of an adverse outcome than accepting the medication [26]. *Belief bias* [80] was assessed using four syllogistic reasoning problems that conflict with the empirical and the logical status of the conclusion, and the total score was calculated as a proportion of answers indicating that participants based their judgments about the conclusion’s validity on the conclusion’s believability [81]. *Commitment bias* [82] was indicated by a preference to continue advocating the health benefits of one food product over another despite the information on new evidence showing no difference between them.

Within the subset of sociopolitical beliefs, we measured religiosity and spirituality using single items rated on a 5-point scale (ranging from 1 –*completely disagree*, to 5 –*completely agree*) and political orientation rated on a 7-point scale (1 –*far left*; 4 –*center*; 7 –*far right*). In addition, we assessed healthcare-related beliefs and experiences, namely, trust in the healthcare system and professionals, negative experiences with the healthcare system, and trust in science.

Trust in the healthcare system and professionals was assessed by four gender-neutral items from the *Women’s trust and confidence in healthcare system—WITCH scale* [83]. Two items with the highest loadings from the *Particularized (interpersonal) trust* dimension were used to assess trust in healthcare professionals, while two items with the highest loadings from the *Generalized (mis)trust* dimension assessed trust in the healthcare system, and all items were rated on a 5-point scale (ranging from 1 –*completely disagree*, to 5 –*completely agree*). *Negative experiences with the healthcare system* were assessed using five items (ranging from 1 –*never*, to 5 –*every time*) with high loadings from the experiences with the medical system scale [84]. *Trust in science* was assessed with two items assessing trust in scientific achievements and belief that the scientific method is the best way to reach the truth.

### Analytical strategy

**Fig 1:**
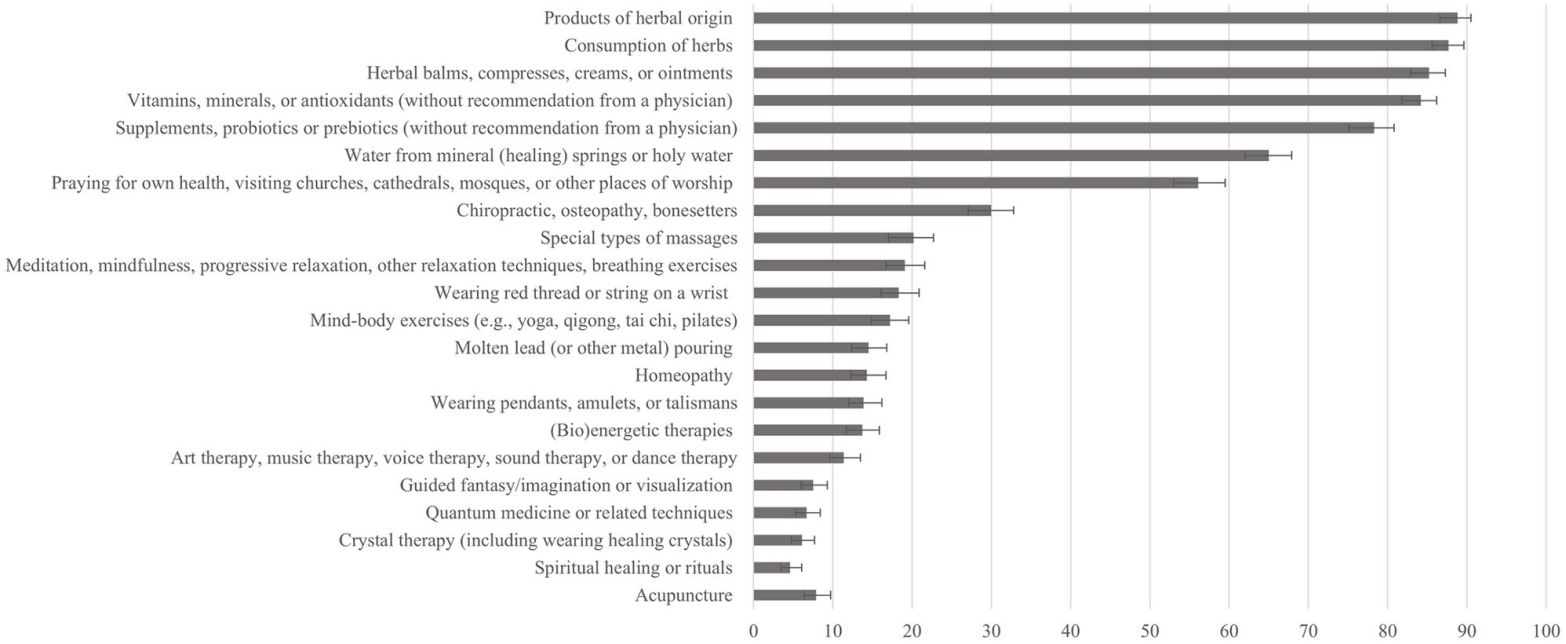
Lifetime prevalence of different TCAM practices. Confidence intervals are calculated using Wilson’s procedure [89].

**Fig 2:**
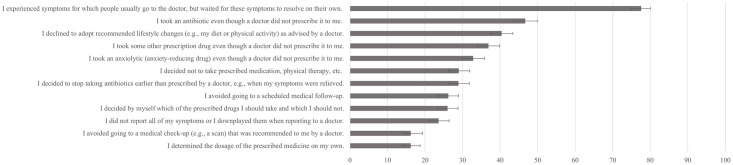
Lifetime prevalence of different iNAR behaviors. Confidence intervals are calculated using Wilson’s procedure.

Preliminary analysis included descriptive statistics and correlations between all measured variables. The prevalence of TCAM and iNAR behaviors was calculated as the proportion of individuals in the sample who have engaged in these two types of behaviors. We also present the percentage of individuals engaging in each specific TCAM and iNAR behavior (complete list in Figs 1 and 2, and S2 Table), alongside Cronbach alpha reliabilities for all multi-item measures. In line with the preregistration, we tested eight mediation models, four with Disintegration as the predictor and four with CRT as the predictor of TCAM, using apophenia belief in conspiracy theories (both general and medical), magical beliefs about health, and superstitiousness as mediators [56].

We also regressed both questionable practices onto six blocks of predictors in two hierarchical linear regression analyses. These sets contain all variables described in the protocol paper, namely, sociodemographics, health-related variables, distal predictors including personality and thinking styles variables, proximal predictors including irrational mindset and cognitive biases, sociopolitical variables, and healthcare-related beliefs and experiences. In addition, we regressed the TCAM domains onto the same predictor sets.

**Fig 3:**
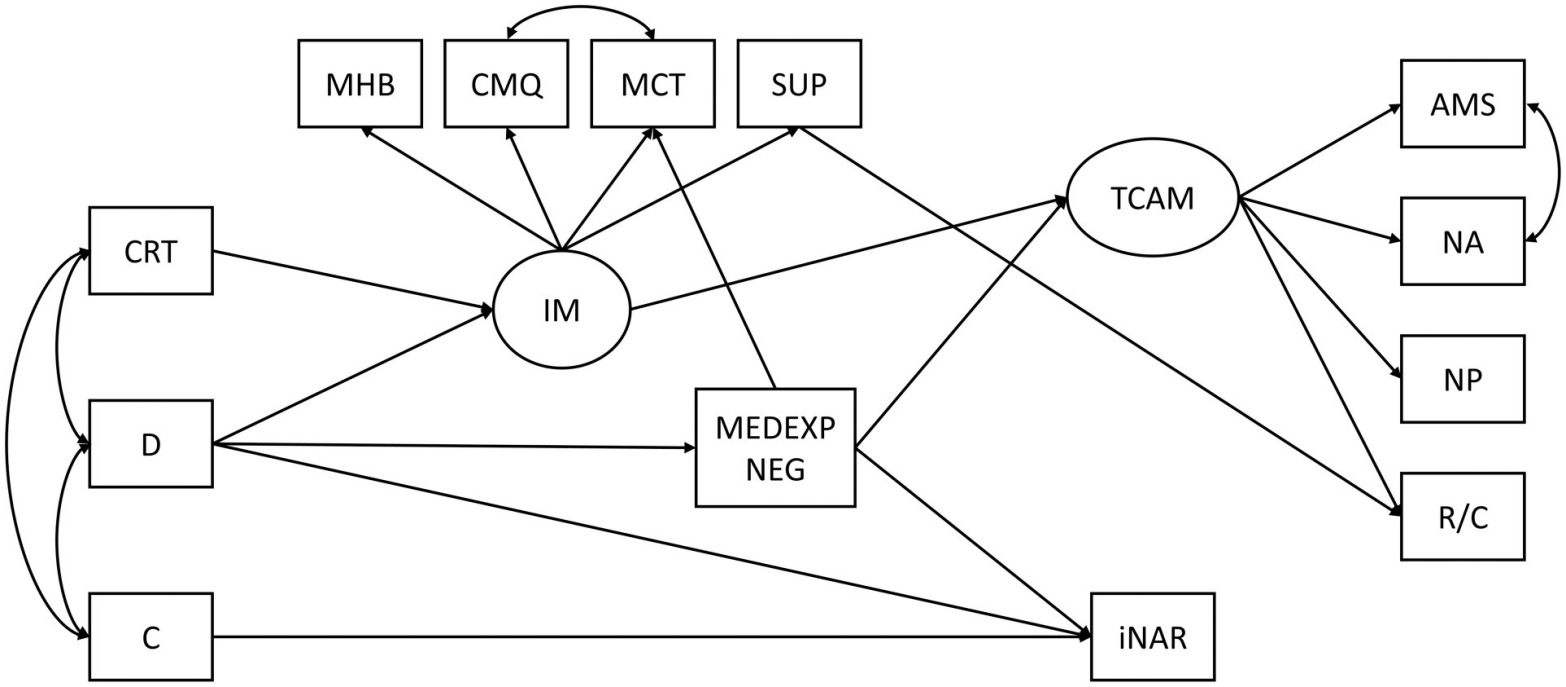
Predictors of the questionable research practices—The initial model, tested by SEM. Manifest variables: CRT—Cognitive reflection; D—Disintegration; H—Honesty/Humility; C—Conscientiousness; MHB—Magical beliefs about health; CMQ—General conspiracy mentality; MCT—Medical conspiracy beliefs; SUP—Superstitiousness; MEDEPXNEG—Negative experiences with the healthcare system; AMS—Alternative medical systems; NA—New Age practices; NP—Natural product-based treatments; R/C—Rituals/Customs. Latent variables: IM—Irrational mindset; INAR—Intentional non-adherence to medical recommendations; TCAM—Use of traditional, complementary and alternative medicine.

As a more rigorous check of predictors of questionable health practices, we tested the most robust previous findings [1, 10] via SEM (see Fig 3). The main assumption instantiated by the model was that the irrational mindset represents the most important proximal correlate of TCAM behavior [56], mediating the relationships between distal predictors and TCAM [10, 34, 85]. Unlike TCAM, we did not expect the irrational mindset to be of critical relevance for iNAR, but that distal predictors—Disintegration, low Honesty and low Conscientiousness will play a more prominent role [10, 15, 34]. We also expected negative experiences with the healthcare system to facilitate medical conspiratory beliefs and use of questionable health practices [15]. A path from Disintegration to negative experiences with the healthcare system was based on our earlier findings that those high on Disintegration tend to report more traumatic experiences [86]. The irrational mindset, as well as TCAM, are modeled as latent variables. The former is indicated through magical beliefs about health, conspiratory beliefs, and superstitiousness, and the latter through the four aforementioned aspects of TCAM. The error covariance was allowed between general and medical conspiracy beliefs (as both are conspiratory beliefs). To evaluate the adequacy of the model, several indices assessing misspecifications are examined: Standardized Root Mean Square Residual (SRMR) to assess structural aspects, and Root Mean Square Error of Approximation (RMSEA), and Comparative Fit Index (CFI) to assess the measurement aspects of a model. Hu and Bentler [87] suggested that RMSEA should be less than .06, CFI should be greater than .95 (although values from .90 can be considered acceptable according to Marsh and colleagues [88]), and SRMR should be less than .08, to evaluate a model as an acceptable one.

### Deviations from the protocol

In the protocol, predictors were classified into three sets: (1) sociodemographic and health-related variables, (2) distal, and (3) proximal predictors. Here we made further differentiation within the first and third set. In the first set, sociodemographic variables were separated from health-related ones. The third set (proximal predictors) was differentiated as the irrational mindset, sociopolitical attitudes, and experiences with/trust in the healthcare system. This refinement was done to increase precision in our conclusions regarding the predictors of TCAM and INAR. We did not include digital literacy and scientistic beliefs in the current analysis, as they will bee a) the focus of a PhD thesis and b) we did not pre-register any hypothesis regarding their role.

## Results

The instruments, anonymized dataset, and supplementary materials are all made publicly available at the Open Science Framework (https://osf.io/gfp4q/?view_only=c28dbf1d04ac4924afbb63deba9ca599).

### Lifetime prevalence of questionable health practices

Lifetime prevalences for specific TCAM practices and iNAR behaviors are presented in Figs 1 and 3, and Supplementary material (S2 Table), while descriptive statistics for all variables are presented in S3 Table.

A typical participant reported using around one-third of listed TCAM practices and one-third of non-adherence behaviors during their lifetime. Among TCAM practices, the most frequent were a) the consumption of herbs or honeybee products, b) products of herbal origin, c) the use of herbal balms, creams, and ointments, and d) the consumption of supplements, all with lifelong prevalences above 75%. Other frequent practices were those related to religion—using water from mineral springs or holy water (65%) or praying for one’s health (56%). Among the least common but still notable TCAM practices were wearing a red thread or string around the hand (18%), molten lead pouring (14%), homeopathy (14%), and crystal therapy (6%). Overall, 99% of participants reported engaging in at least one TCAM practice during their lifetime. Natural products were the most prevalent category, with 97% of individuals having used at least one of these practices, surpassing the use of rituals/customs (75%), and alternative systems (49%), while only 30% reported using any of New Age medicine practices.

The most frequently reported iNAR behaviors were ignoring symptoms that required a visit to a doctor (78%), and taking antibiotics when not prescribed by a doctor (47%). In contrast, the least frequent behaviors included avoiding recommended medical check-ups (16%) and changing the dosage of the prescribed medication without consulting a healthcare professional (16%). A total of 89% of participants reported having engaged in at least one iNAR behavior.

### Psychological predictors of questionable health behaviors

The full correlation table is presented in the Supplementary material (S4 Table). Due to a large number of intercorrelating predictors, the interpretation of their raw correlations with health-related behavior is not particularly informative. Therefore, the focus will be on the results of the regression analyses.

The first—sociodemographic block—was not predictive of iNAR behaviors but it accounted for 9.9% of the variance in TCAM use, with women, those younger, more educated, and living in more urban settlements being more prone to use TCAM practices (Table 1). The second predictor block comprising health-related variables proved to be predictive of both iNAR and TCAM use, accounting for 3.9% and 1.3% of the variance, respectively. In the third block, distal predictors comprising personality traits and thinking dispositions and styles accounted for an additional 5.3% of the variance in iNAR and 6.7% of the variance in TCAM. The fourth block, comprising irrational mindset variables, showed much higher predictive power for TCAM than iNAR. Namely, the irrational mindset accounted for an additional 2.8% of the variance in iNAR, but an additional 7.7% of the variance in overall TCAM use—with proneness to magical health beliefs, beliefs in extra-sensory perception, and lower doublethink predicting a higher TCAM score. Sociopolitical attitudes and beliefs entered in the fifth block, were incrementally predictive of TCAM only (explaining 2.1% of the variance in TCAM use), with spirituality significantly contributing to the prediction. Finally, within the last block, only negative experiences with the healthcare system were a significant incremental predictor, accounting for 4.6% and 2.2% of the variance in iNAR and TCAM, respectively. In general, TCAM proved to be consistently more strongly related to psychological variables, especially to the irrational mindset and sociopolitical attitudes and beliefs, while iNAR showed somewhat higher correlations with the experiences with the healthcare system.

**Table 1:** Hierarchical regressions with iNAR, TCAM, and its aspects as outcome variables.

		iNAR	TCAM	Alternative medical systems	New Age	Natural products	Rituals/Customs
	Predictors	β	β	β	β	Β	β
Block 1	Female gender	-.02	.09**	.01	.07	.06	.09**
	Age	-.03	-.16***	.05	-.20***	-.07	-.17***
	Rural	.00	-.14***	-.16***	-.11***	-.04	-.05
	Education	.02	.09**	.11**	.05	.01	.06
	Socio-economic status	.08	.06	.11**	.03	.05	-.03
	ΔF(5, 997)	0.92	21.82***	12.37***	21.03***	5.83***	13.31***
	ΔR^2^	0.5%	9.9%	5.8%	9.5%	2.8%	6.3%
Block 2	BMI	.05	-.01	-.02	-.04	.07	-.04
	Smoking	-.07	-.04	-.02	-.03	-.06	-.01
	Self-reported health	-.03	.00	-.04	.01	.02	.01
	Chronic illnesses (number)	.05	.07	.09*	.03	.05	.02
	ΔF(4, 993)	10.00***	3.52**	3.84**	3.14	2.67	1.94
	ΔR^2^	3.9%	1.3%	1.4%	1.1%	1.0%	0.7%
Block 3	Honesty-Humility	-.06	-.10**	-.09	-.09**	-.06	-.03
	Emotionality	.02	.05	-.06	.02	.09	.07
	Extraversion	-.08	.02	.06	.04	-.07	.01
	Agreeableness	-.05	.03	.00	-.01	.08	.02
	Conscientiousness	-.08	.02	.01	.00	.05	.00
	Openness	-.05	.08	.05	.12**	.08	-.04
	Disintegration	.06	.06	.04	.08	-.02	.08
	REI rational	.05	-.02	.04	-.03	.01	-.02
	REI experiential	.02	.05	.10**	.04	-.05	.02
	AOT	.03	.05	.05	.01	.09	-.01
	CRT	-.01	-.03	.01	-.04	-.03	-.01
	ΔF(11, 982)	5.19***	7.27***	4.75***	7.35***	3.16***	6.78***
	ΔR^2^	5.3%	6.7%	4.7%	6.8%	3.3%	6.6%
Block 4	CMQ	.04	-.01	-.05	-.03	.06	.01
	MCT	.00	.04	.02	.02	.06	.00
	Superstitiousness	-.04	-.01	-.08	-.02	-.02	.09**
	MHB	-.03	.13**	.11	.11**	.02	.10
	ESB	.03	.11**	.08	.04	.07	.08
	Doublethink	-.03	-.14***	-.07	-.15***	-.06	-.08
	GABS	.00	-.07	-.03	-.07	.00	-.07
	Illusory correlation	-.06	.03	.03	.04	-.02	.02
	Naturalness bias	-.02	-.01	.00	-.04	.02	.01
	Belief bias	-.04	.06	.08	.00	.07	.01
	Omission bias	.00	.04	.03	.04	.01	.03
	Commitment bias	.09**	-.04	-.04	-.01	.02	-.07
	ΔF(12, 970)	2.63**	8.31***	3.48***	5.48***	2.60**	7.28***
	ΔR^2^	2.8%	7.7%	3.6%	5.2%	2.9%	7.1%
Block 5	Religiosity	-.05	.07	-.05	-.08**	.01	.29***
	Spirituality	.02	.10**	.05	.18***	-.02	.05
	Political orientation	.06	.07	.09**	.00	.07	.04
	ΔF(3, 967)	1.45	9.49***	3.13	11.61***	1.45	37.06***
	R^2^	0.4%	2.1%	0.8%	2.7%	0.4%	8.2%
Block 6	Distrust in med. system	.05	-.01	-.04	.02	.04	-.03
	Trust in medical staff	-.05	.03	.01	.01	.05	.02
	Trust in science	-.01	-.03	-.05	-.04	.03	.00
	Negative experiences with the healthcare system	.19***	.17***	.18***	.14***	.08	.05
	ΔF(4, 963)	13.27***	7.69***	8.29***	6.03***	1.76	0.69
	ΔR^2^	4.6%	2.2%	2.8%	1.8%	0.7%	0.2%
Overall	F(39, 963)	5.19***	10.51***	5.86***	9.23***	3.08***	10.12***
	R^2^	17.4%	29.8%	19.2%	27.2%	11.1%	29.1%

Note.

***p < .001,

** *p* < .01, TCAM–traditional, complementary, and alternative medicine; iNAR–intentional nonadherence to official medical recommendations; BMI—Body mass index; AOT—Actively Open-Minded Thinking; CRT—Cognitive reflection; CMQ—Conspiracy mentality; MCT—Medical conspiracy theories; MHB—Magical health beliefs; ESB—Extrasensory beliefs; GABS—General Attitude and Belief Scale.

Regarding the domains of TCAM practices, the use of natural product-based practices appears to be less similar to the other three factors in terms of the structure of their psychological predictors (see Table 1). Namely, it is the least related to psychological, but also other predictors included in the study. Sociopolitical attitudes, distal predictors, irrational mindset, and sociodemographic variables explained more variance in New Age medicine and Rituals/Customs than in the other two TCAM domains. Use of New Age medicine is related to spirituality and low religiosity, reflecting higher Openness of those who use it, while those using Alternative systems and Rituals/Customs appear to be more conservative and religious. Negative experiences with the healthcare system were related only to the use of Alternative medical systems and New Age medicine, and explained a similar amount of variance in both of these domains.

Our expectations regarding the mediating effects of apophenia, belief in conspiracy theories (general and specific-medical), superstitiousness, and magical health beliefs on the relationship between Disintegration and TCAM, as well as between cognitive reflection and TCAM, were partly confirmed. In all models, Disintegration had a positive, while cognitive reflection had a negative contribution to TCAM. However, only belief in medical conspiracy theories and magical beliefs about health were significant mediators of these relationships (see S2 Fig), whereas we observed no mediating effects of apophenia, conspiracy mentality, or superstitiousness. For cognitive reflection, there was a full mediation in both cases. For Disintegration, we observed full mediation for magical beliefs about health and partial mediation for medical conspiracies.

As for the tested SEM model, only the CFI was slightly below the acceptable level (χ^2^_(df)_ = 254.16_(63)_, RMSEA_(90% CI)_ = .055_(.048–.062)_, SRMR = .049, CFI = .891). Due to skewed distribution of some of the variables a maximum likelihood estimator with robust standard errors (MLR) was used. The paths were double standardized (the variances of the continuous latent variables as well as the variances of the background and outcome variables were used for standardization).

Firstly, the assumption of the incremental importance of Honesty in questionable health practices was not empirically supported in this sample. It was also evident that there are relationships between Alternative medical systems and New Age medicine beyond the general TCAM factor. Namely, both imply a more elaborated, alternative conceptual/ideological structure in which the doctrine of holistic approach plays a significant role. Additionally, as our regression analyses suggest, this correlation might also reflect negative experiences with the healthcare system that do not characterize the remaining two TCAM domains.

**Fig 4:**
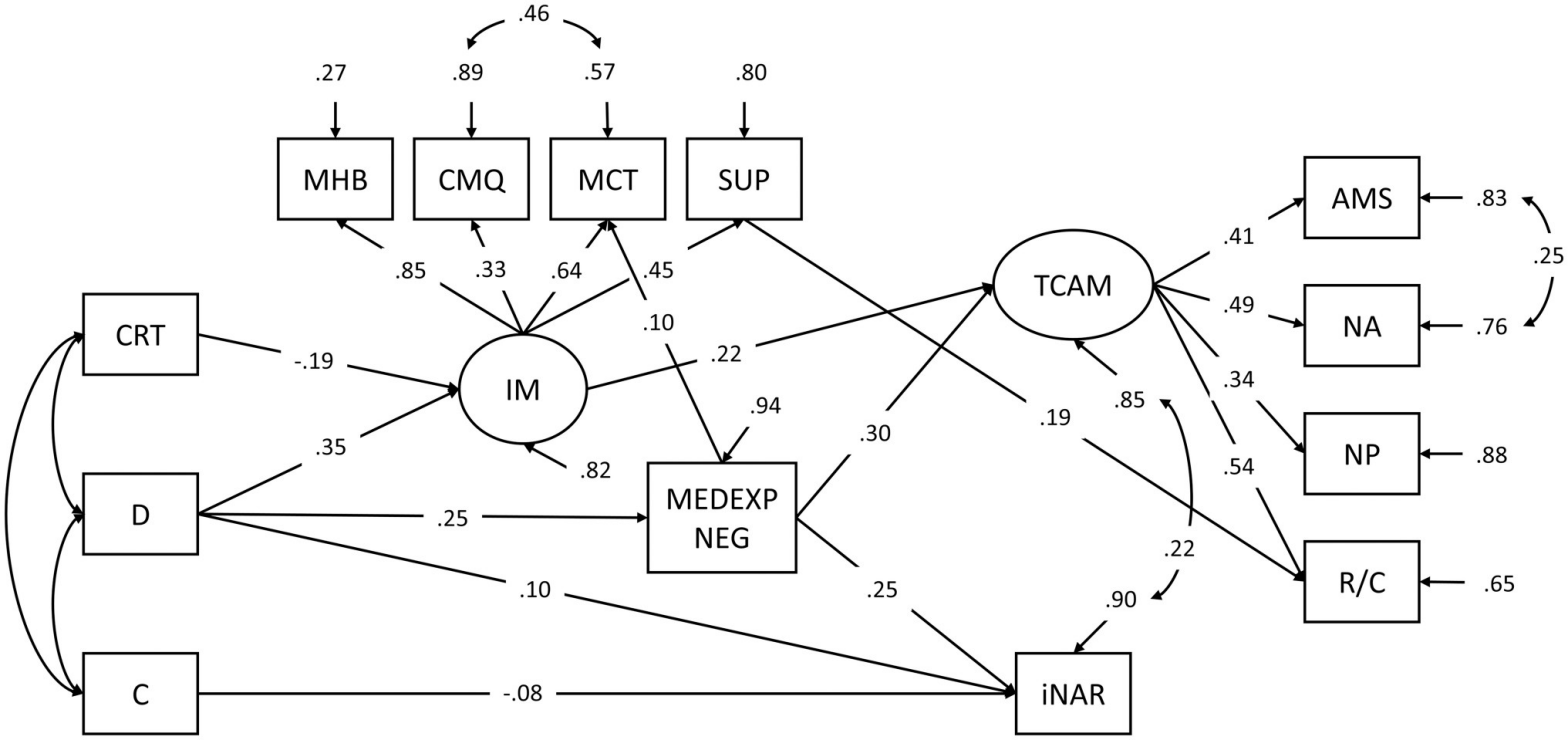
Predictors of the questionable research practices—The final SEM model. Manifest variables: CRT—Cognitive reflection; D—Disintegration; C—Conscientiousness; MHB—Magical beliefs about health; CMQ—General conspiracy mentality; MCT—Medical conspiracy beliefs; SUP—Superstitiousness; MEDEPXNEG—Negative experiences with the healthcare system; AMS—Alternative medical systems; NA—New Age practices; NP—Natural product-based treatments; R/C—Rituals/Customs. Latent variables: IM—Irrational mindset; INAR—Intentional non-adherence to medical recommendations; TCAM—Use of traditional, complementary and alternative medicine. Path coefficients are indicated by arrows between variables. Residual variances of the predicted variables are marked by inward oriented arrows. All shown paths were statistically significant.

After excluding Honesty from the model and introducing the error covariance between the two TCAM domains, the model (Fig 4) had acceptable fit indices: (χ^2^_(df)_ = 215.33_(54)_, RMSEA_(90% CI)_ = .055_(.047–.062)_, SRMR = .047, CFI = .908). As expected, irrational beliefs play a crucial role in TCAM, mediating its relationships with Disintegration and low cognitive reflection, as distal predictors. For iNAR, the effects of Disintegration and low Conscientiousness on it were expectedly not mediated by the irrational mindset. However, a part of the statistical effect of Disintegration seems to be mediated via negative experiences with the healthcare system. Moreover, negative experiences with the healthcare system were related to both types of questionable practices. We consider the relationships captured by this model—which also incorporates the findings from the mediation analyses—the most empirically robust, theoretically grounded, and likely generalizable across various samples.

## Discussion

We observed that both types of questionable health practices were more of a norm than an exception in the general population. Namely, only 8.7% of participants reported that they had never engaged in iNAR, while a minuscule 0.8% stated the same for TCAM.

Such widespread prevalence was not reported in previous studies which found TCAM use to be around 25% [14]. This discrepancy is due to important methodological differences. First, our study assessed lifetime TCAM prevalence rather than the prevalence in shorter time frames (e.g. in the past year). Also, we have included the use of various herbal products, vitamins, and supplements for health-related purposes, while previous studies typically more narrowly defined TCAM as visits to TCAM providers. The overwhelming prevalence of herbal products use in our sample (97.8%) is, in fact, in line with a systematic review of TCAM use in the EU that found use of herbal products to be the most frequent type of TCAM [90], with fairly high percentages of current use reported in the USA [91] and also overwhelmingly positive representation of herbal treatments in Serbian online media [12]. Somewhat more surprising was the finding that over 75% of the sample used rituals/customs, with around a third of participants using at least one of the local, traditional practices—wearing pendants, amulets, or talismans, wearing red thread/string around the wrist, and molten lead pouring.

Regarding iNAR, notably and in line with previous findings [11, 92–95], almost half of the population used antibiotics without a prescription, and a third did the same with anxiolytics. More than three quarters of the population experienced symptoms for which people usually visit physicians, but waited for these symptoms to subside. Finally, we replicated the finding that people engaging in TCAM also tend to intentionally non-adhere to medical recommendations (iNAR) [10, 15, 20].

Consistent with previous studies [10, 15] (also see review [54]), women, more educated individuals, and those from larger towns/cities were more prone to engage in TCAM. This profile especially characterized those practicing New Age TCAM. Still, psychological variables were of greater importance in explaining questionable health practices. As expected [15, 17–21], the irrational mindset, most notably magical health beliefs and belief in medical conspiracy theories, was the most important psychological factor of TCAM. These irrational beliefs mediated the relationship between higher Disintegration/lower cognitive reflection and questionable health behaviors. For iNAR, on the other hand, distal factors—high Disintegration and low Conscientiousness—were the most relevant (in line with previous studies [10, 15]), whilst negative experiences with the healthcare system turned out to be important in facilitating both types of questionable health practices.

The whole predictor set was more successful in explaining TCAM, compared to iNAR (30% vs. 17%, respectively). The use of on-evidence based alternative medical practices thus seem to be more dependent on psychological factors. This was especially true for the practices which are more ideologically charged, such as Alternative medical systems and New Age medicine. Conversely, intentional non-adherence to medical recommendations was predominantly embedded in negative experiences with the healthcare system. All of these findings have been observed in our previous studies on community samples [10, 15].

However, this picture becomes more nuanced when we consider the larger set of factors. While it seems that individuals who use TCAM practices tend to have a more conservative worldview, i.e. that they are more prone to right-wing political ideology and religiosity, they also tend to be more open-minded and less prone to doublethink. Their religiousness might, therefore, be more the result of a spiritual quest than a manifestation of conservatism, which is especially visible among those adhering to New Age medicine. Furthermore, unlike those inclined to endorse irrational beliefs, individuals who use TCAM (especially New Age practices and rituals/customs) tend to be younger [15]. It seems that although the individuals prone to irrational beliefs tend to be less open-minded [96] and older, those practicing TCAM—despite irrational beliefs being the strongest predictor of these practices—were more open-minded and younger. The robustness of these fine differences, however, needs to be further tested independently.

Somewhat surprisingly, our data provides no evidence that health status is related to TCAM while it was only weakly related to iNAR behaviors. It seems that, when they appear, health-related problems only trigger the behavioral tendencies that are already part of a person’s psychological repertoire. This could also partly be due to the fact that we measured lifetime prevalence, and not the short-term frequency of both types of behaviors. We opted for such measurement to ensure we will capture those behaviors in the general population, but finer differences regarding the health status might emerge in specific populations (with chronic diseases, for example) and in a shorter time frame.

### Implications

There are two central findings of this study: a) intentional non-adherence and TCAM are widespread; b) psychological factors such as irrational beliefs, personality traits, and thinking styles in health choices suggest their deep psychological roots. Bearing in mind the potentially harmful consequences of these practices, there is a need to find a functional and efficient approach to promote evidence-based health behavior.

For medical professionals, our results imply that they should expect that patients may try alternative recommendations instead of being fully adherent to treatment. The role of negative experiences with the healthcare system in both types of questionable health practices, especially iNAR, indicates that the quality of the physician-patient relationship is a crucial protective factor against questionable health behaviors. Training for clinicians about the prevalence and types of non-adherence and TCAM, as well as the importance of patient-clinician relationships in establishing adherence, should be incorporated in future public health policies.

Our results have important implications to public health communication, as well. Although we find that TCAM is rooted in irrational beliefs which are notoriously hard to change (we even include resistance to disconfirming evidence in their definition), there are still some promising interventions that could be tested to this end. That could be paradoxical, extreme attitude-consistent messages [97], myth-debunking, e.g. the myth of profit-oriented, unscrupulous “Big Pharma’’ versus non-profit, moral CAM industry [19, 98], inoculation interventions [99], or narrative interventions [100].

Finally, institutional stakeholders should interpret the widespread use of TCAM as a signal to tighten regulations in testing, distributing/selling these products and services, as well as their promotional claims of safety and efficacy, and in media reporting (WHO guidelines; also Lazić and colleagues [12]). Certain nudges (choice architecture, increased pricing, marketing) in support of evidence-based practices could also be considered.

### Limitations

The sample in our study deviated from the population quotas in certain aspects, particularly in terms of education and region. We thus had to use weights to obtain precise lifetime prevalence estimates of questionable health practices.

We sampled the general population and did not specifically focus on patients. The sample therefore did not allow a breakdown by specific chronic illnesses, to study specific subpopulations such as, for example, cancer patients. A targeted approach to patients when they first enter the official healthcare system and tracking down their pathway to care in a case-control study design would provide more precise information on their way of TCAM use.

To be able to measure a wide range of constructs, we mostly used their short forms or even single-item assessments. This led to comparatively lower internal consistencies, which may, in turn, have attenuated the true correlations between variables. Due to the same constraints, we assessed illusory pattern perception via only one test (albeit demanding for participants). Recent research, however, suggests that multiple cognitive tests are needed to capture sufficient commonality for it to be reliably measured [41]. This could be a reason we did not find the expected relationships with either Disintegration or any irrational beliefs.

The current study provided correlational evidence which should not be taken as evidence on unidirectional causality. Although it conceptually makes sense to assume causality from dispositions to behavior, the backward iterative loops from behavior to dispositions (especially beliefs and attitudes) are also possible: longitudinal studies would be informative about the direction of the relationship between the groups of factors and iNAR/TCAM behaviors. Additionally, experiments should test the proposed impact of irrational beliefs on these behaviors.

The relationships between variables captured by our SEM model were remarkably robust in our recent line of studies [10, 15]. However, other relationships suggested here by the regression models are yet to be replicated. In addition, bearing in mind that most of the findings have been detected on the Serbian population, to confirm their generalizability, it is important to investigate their invariance across cultures.

## Conclusions

Our results compellingly demonstrate how widespread questionable health practices are in the general population of Serbia, and provide useful prevalence estimates for specific types of non-adhering and TCAM behaviors. We once again show the two types of behaviors to be positively related, but not strongly, which is not surprising considering the difference in the profiles of variables predicting them. As we expected, psychological variables seem to be of central importance (more than socio-economic and health status), especially for TCAM. The most important proximal factors of TCAM use were irrational beliefs, which can be traced further down to a basic personality trait—Disintegration—and to a cognitive disposition manifesting itself in the lower level of reflection. iNAR was predicted by negative experiences with the healthcare system, but also by personality traits high Disintegration and low Conscientiousness. Taken together, our results suggest that promoting adherence to evidence-based health practices likely requires more than just presenting individuals with factual information and minimizing negative experiences with the healthcare system (albeit those are necessary first steps). For such policy to be effective, it needs to address irrational beliefs and biased reasoning shown to enable and maintain the questionable ones.

## Supporting information

S1 Table Demographic distribution comparison: Sample vs. population. (DOCX)

S2 Table Percentage of participants reporting given behavior or practice (N = 1003). (DOCX)

S3 Table Descriptives for all variables. (DOCX)

S4 Table Correlations between variables. (DOCX)

S1 Fig Density plots suggesting careless responding of those with maximum scores. (TIF)

S2 Fig Mediation analysis. Disintegration and cognitive reflectivity as predictors, magical beliefs about health, general conspiracy mentality and medical conspiracy beliefs as mediators. CRT—Cognitive reflection; DELTA—Disintegration; MBH—Magical beliefs about health; CMQ—General conspiracy mentality; MCT—Medical conspiracy beliefs; TCAM—Use of traditional, complementary and alternative medicine.
(TIF)
