# Supplementary material for: Lifetime prevalence of questionable health behaviors and their psychological roots: A preregistered nationally representative survey
Source: PLoS One. 2024 Nov 6;19(11):e0313173. doi: 10.1371/journal.pone.0313173 (PMC11540216; doi:10.1371/journal.pone.0313173)
Supplement: S1 Table — (DOCX) [file pone.0313173.s001.docx]

**S1 Table. Demographic Distribution Comparison: Sample vs. Population**

|  |  | Sample (weighted) | | Population | |
| --- | --- | --- | --- | --- | --- |
|  |  | f | % | f | % |
| Sex | Male | 502 | 50.0 | 2739739 | 48.1 |
|  | Female | 501 | 50.0 | 2951812 | 51.9 |
| Age | 18-29 | 183 | 18.3 | 848012 | 15.4 |
|  | 30–39 | 168 | 16.7 | 853209 | 15.5 |
|  | 40–49 | 162 | 16.1 | 948051 | 17.3 |
|  | 50–59 | 189 | 18.9 | 901987 | 16.4 |
|  | 60–69 | 147 | 14.7 | 974046 | 17.7 |
|  | 70–74 | 154 | 15.4 | 434378 | 7.9 |
|  | 75+ | 0 | 0 | 532337 | 9.7 |
| Education | No formal education | 29 | 2.9 | 357406 | 6.3 |
|  | Completed elementary school | 121 | 12.1 | 1013067 | 17.9 |
|  | Completed secondary school (three years) | 118 | 11.7 | 990569 | 17.5 |
|  | Completed secondary school (four years) | 392 | 39.1 | 1735465 | 30.6 |
|  | Completed gymnasium | 92 | 9.2 | 228669 | 4.0 |
|  | Completed University college | 85 | 8.5 | 410311 | 7.2 |
|  | Completed University - Bachelor’s or higher | 166 | 16.5 | 933340 | 16.5 |
| Region | Belgrade | 232 | 23.1 | 1427135 | 25.1 |
|  | Vojvodina | 270 | 26.9 | 1485259 | 26.1 |
|  | Šumadija and Western Serbia | 284 | 28.3 | 1561084 | 27.4 |
|  | Southern and Eastern Serbia | 218 | 21.8 | 1218073 | 21.4 |

*Note*. The 2022 census data in this table are available from The Statistical Office of the Republic of Serbia. For more detailed information, please refer to the official website: <https://www.stat.gov.rs/>.
