# Supplementary material for: Lifetime prevalence of questionable health behaviors and their psychological roots: A preregistered nationally representative survey
Source: PLoS One. 2024 Nov 6;19(11):e0313173. doi: 10.1371/journal.pone.0313173 (PMC11540216; doi:10.1371/journal.pone.0313173)
Supplement: S2 Table — (DOCX) [file pone.0313173.s002.docx]

**S2 Table. Percentage of participants reporting given behavior or practice (N = 1003)**

| **Items** | % | 95% CI |
| --- | --- | --- |
| **TCAM** |  |  |
| Acupuncture | 7.9 | 6.3 - 9.7 |
| Homeopathy | 14.3 | 12.3 - 16.6 |
| Quantum medicine or related techniques | 6.7 | 5.3 - 8.4 |
| (Bio)energetic therapies | 13.7 | 11.7 - 15.9 |
| Crystal therapy (including wearing healing crystals) | 6.1 | 4.8 - 7.8 |
| Chiropractic, osteopathy, bonesetters | 30.0 | 27.2 - 32.9 |
| Special types of massages (including shiatsu, hot stone massage,  marma point massage, meridian massage therapy, tui na, rolfing massage, hydrotherapy, Kneipp therapy, Trigger-point massage, lymphatic drainage massage, etc.) | 20.2 | 17.8 - 22.8 |
| Mind-body exercises (e.g., yoga, qigong, tai chi, pilates) | 17.2 | 14.9 - 19.6 |
| Spiritual healing or rituals | 4.6 | 3.5 - 6.1 |
| Art therapy, music therapy, voice therapy, sound therapy, or dance therapy | 11.4 | 9.6 - 13.5 |
| Meditation, mindfulness, progressive relaxation, other relaxation techniques, breathing exercises | 19.1 | 16.8 - 21.6 |
| Guided fantasy/imagination or visualization | 7.5 | 6.0 - 9.3 |
| Products of herbal origin (e.g., teas, drinks, extracts, drops, essences, tinctures made of plants such as comfrey, chamomile, ginger, garlic, aloe vera, etc.) | 88.8 | 86.7 - 90.6 |
| Herbal balms, compresses, creams, or ointments (e.g., garlic, marigold, lavender, yarrow, olive oil) | 85.2 | 82.9 - 87.3 |
| Consumption of herbs (e.g., garlic, houseleek, dried figs, cornel) or honeybee products (honey, honey bee pollen, honeycomb, propolis) | 87.7 | 85.5 - 89.6 |
| Vitamins, minerals, or antioxidants (without recommendation from a physician) | 84.2 | 81.8 - 86.3 |
| Supplements, probiotics or prebiotics (without a recommendation from a physician) | 78.3 | 75.7 - 80.8 |
| Wearing pendants, amulets, or talismans | 13.9 | 11.9 - 16.2 |
| Wearing red thread or string on a wrist | 18.3 | 16.1 - 20.8 |
| Molten lead (or other metal) pouring | 14.5 | 12.4 - 16.8 |
| Water from mineral (healing) springs or holy water | 65.0 | 62.0 - 67.9 |
| Praying for own health, visiting churches, cathedrals, mosques, or other places of worship | 56.1 | 53.0 - 59.2 |
| **iNAR** |  |  |
| I experienced symptoms for which people usually go to the doctor, but waited for these symptoms to resolve on their own instead of going to the doctor. | 77.6 | 74.9 - 80.1 |
| I did not report all of my symptoms or I downplayed them when reporting to a doctor. | 23.6 | 21.1 - 26.4 |
| I took an antibiotic even though a doctor did not prescribe it to me. | 46.7 | 43.7 - 49.8 |
| I took an anxiolytic (anxiety-reducing drug) even though a doctor did not prescribe it to me. | 32.8 | 30.0 - 35.8 |
| I took some other prescription drug even though a doctor did not prescribe it to me. | 36.9 | 34.0 - 39.9 |
| I decided to stop taking antibiotics earlier than prescribed by a doctor, e.g., when my symptoms were relieved. | 28.9 | 26.2 - 31.8 |
| I decided not to take prescribed medication, physical therapy, etc. | 29.0 | 26.3 - 31.9 |
| I determined the dosage of the prescribed medicine on my own. | 16.2 | 14.1 - 18.6 |
| I decided by myself which of the prescribed drugs I should take and which I should not. | 26.0 | 23.4 - 28.8 |
| I avoided going to a medical check-up (e.g., a scan) that was recommended to me by a doctor. | 16.2 | 14.1 - 18.6 |
| I avoided going to a scheduled medical follow-up. | 26.2 | 23.5 - 29.0 |
| I declined to adopt recommended lifestyle changes (e.g., my diet or physical activity) as advised by a doctor. | 40.4 | 37.4 - 43.5 |

*Note*. TCAM – traditional, complementary, and alternative medicine, iNAR – intentional nonadherence to official medical recommendations.

Confidence intervals are calculated using Wilson’s procedure (1927).
