# Supplementary material for: Lifetime prevalence of questionable health behaviors and their psychological roots: A preregistered nationally representative survey
Source: PLoS One. 2024 Nov 6;19(11):e0313173. doi: 10.1371/journal.pone.0313173 (PMC11540216; doi:10.1371/journal.pone.0313173)
Supplement: S3 Table — (DOCX) [file pone.0313173.s003.docx]

**S3 Table. Descriptives for all variables**

|  | M | SD | No. of items | Reliability |
| --- | --- | --- | --- | --- |
| Health variables |  |  |  |  |
| BMI | 25.37 | 4.10 | 1 | / |
| Smoking | Yes (n = 352; 35.1%), No (n = 651, 64.9%) | | | |
| Health status | 3.76 | 0.91 | 1 | / |
| Number of chronic illness | 0.99 | 1.26 | 1 | / |
| Presence of chronic illness | At least one (n = 528, 52.6%), None (n = 475, 47.4%) | | | |
| Health behaviors |  |  |  |  |
| TCAM overall | 0.34 | 0.15 | 22 | .77 |
| Alternative systems | 0.15 | 0.21 | 6 | .60 |
| New Age medicine | 0.11 | 0.21 | 6 | .74 |
| Natural products | 0.85 | 0.25 | 5 | .70 |
| Rituals/Customs | 0.34 | 0.27 | 5 | .64 |
| iNAR | 0.33 | 0.24 | 12 | .76 |
| Distal predictors |  |  |  |  |
| Honesty-Humility | 3.61 | 0.66 | 10 | .74 |
| Emotionality | 3.18 | 0.65 | 10 | .71 |
| eXtraversion | 3.23 | 0.63 | 10 | .76 |
| Agreeableness | 3.18 | 0.60 | 10 | .69 |
| Conscientiousness | 3.59 | 0.62 | 10 | .74 |
| Openness | 3.18 | 0.82 | 10 | .78 |
| Disintegration | 2.40 | 0.71 | 20 | .89 |
| REI-R | 3.54 | 0.97 | 4 | .80 |
| REI-E | 3.50 | 0.80 | 4 | .72 |
| AOT | 3.99 | 0.83 | 8 | .75 |
| CRT | 0.36 | 0.37 | 3 | .67 |
| Proximal predictors |  |  |  |  |
| Apophenia | 4.07 | 3.31 | 12 | .82 |
| Belief in general conspiracy theories | 3.98 | 0.75 | 5 | .76 |
| Belief in medical conspiracy theories | 3.38 | 0.96 | 5 | .83 |
| Magical health beliefs | 3.20 | 0.81 | 10 | .84 |
| Superstitiousness | 2.62 | 0.95 | 5 | .70 |
| Extrasensory perception beliefs | 3.16 | 0.80 | 6 | .76 |
| Doublethink | 6.08 | 2.51 | 11 | .65 |
| GABS | 3.27 | 0.66 | 6 | .63 |
| Social beliefs |  |  |  |  |
| Political orientation | 4.41 | 1.79 | 1 | / |
| Religiousness | 3.29 | 1.33 | 1 | / |
| Spirituality | 2.39 | 1.22 | 1 | / |
| Cognitive biases |  |  |  |  |
| Overconfidence | 48.92 | 35.42 | 1 | / |
| Illusory correlation | 0.44 | 0.50 | 1 | / |
| Naturalness bias | 0.76 | 0.43 | 1 | / |
| Omission bias | 0.15 | 0.36 | 1 | / |
| Belief bias | 0.80 | 0.27 | 4 | .61 |
| Commitment bias | 0.48 | 0.50 | 1 | / |
| Healthcare-related beliefs and experiences |  |  |  |  |
| Mistrust in a medical system | 3.60 | 0.92 | 2 | .44 |
| Trust in medical staff | 3.27 | 0.98 | 2 | .75 |
| Negative exp. medical system | 2.09 | 0.79 | 5 | .79 |
| Trust in science | 3.60 | 0.88 | 2 | .71 |
|  |  |  |  |  |

*Note.* BMI - Body mass index; TCAM – traditional, complementary, and alternative medicine; iNAR – intentional nonadherence to official medical recommendations; REI-R - Rational thinking style; REI-E - Experiential thinking style; AOT - Actively Open-Minded Thinking; CRT - Cognitive reflection; GABS - General Attitude and Belief Scale.
