# Supplementary material for: Lifetime prevalence of questionable health behaviors and their psychological roots: A preregistered nationally representative survey
Source: PLoS One. 2024 Nov 6;19(11):e0313173. doi: 10.1371/journal.pone.0313173 (PMC11540216; doi:10.1371/journal.pone.0313173)
Supplement: S4 Table — (DOCX) [file pone.0313173.s004.docx]

**S4 Table. Correlations between variables**

|  | 1 | 2 | 3 | 4 | 5 | 6 | 7 | 8 | 9 | 10 | 11 | 12 | 13 | 14 |
| --- | --- | --- | --- | --- | --- | --- | --- | --- | --- | --- | --- | --- | --- | --- |
| **Socio-demographics** |  |  |  |  |  |  |  |  |  |  |  |  |  |  |
| 1. Gender |  |  |  |  |  |  |  |  |  |  |  |  |  |  |
| 2. Age | -.13^***^ |  |  |  |  |  |  |  |  |  |  |  |  |  |
| 3. Education | .03 | -.26^***^ |  |  |  |  |  |  |  |  |  |  |  |  |
| 4. SES | -.06 | -.14^***^ | .25^***^ |  |  |  |  |  |  |  |  |  |  |  |
| **Health variables** |  |  |  |  |  |  |  |  |  |  |  |  |  |  |
| 5. BMI | -.23^***^ | .19^***^ | -.02 | -.08^**^ |  |  |  |  |  |  |  |  |  |  |
| 6. Smoking | .05 | -.06 | .04 | .06 | .04 |  |  |  |  |  |  |  |  |  |
| 7. Health status | -.03 | -.35^***^ | .19^***^ | .27^***^ | -.14^***^ | .04 |  |  |  |  |  |  |  |  |
| 8. Number of illnesses | .02 | .42^***^ | -.17^***^ | -.16^***^ | .13^***^ | -.03 | -.55^***^ |  |  |  |  |  |  |  |
| **Health behaviors** |  |  |  |  |  |  |  |  |  |  |  |  |  |  |
| 9. TCAM overall | .19^***^ | -.20^***^ | .17^***^ | .07 | -.12^***^ | -.03 | .01 | .00 |  |  |  |  |  |  |
| 10. Alter. Systems | .02 | .00 | .16^***^ | .11^***^ | -.04 | -.03 | -.05 | .09^**^ | .68^***^ |  |  |  |  |  |
| 11. NewAge medicine | .15^***^ | -.25^***^ | .16^***^ | .07 | -.14^***^ | -.04 | .05 | -.08 | .71^***^ | .40^***^ |  |  |  |  |
| 12. Natural products | .11^***^ | -.11^**^ | .09^**^ | .03 | .03 | -.04 | .02 | .01 | .53^***^ | .14^***^ | .11 |  |  |  |
| 13. Rituals/Customs | .21^***^ | -.15^***^ | .04 | -.02 | -.13^***^ | .03 | .01 | -.02 | .67^***^ | .20^***^ | .28^***^ | .20^***^ |  |  |
| 14. iNAR | -.01 | -.06 | .04 | .01 | .06 | -.08^**^ | -.12^***^ | .08 | .20^***^ | .08^**^ | .11^***^ | .19^***^ | .13^***^ |  |

*Correlations between variables (continued)*

|  | 1 | 2 | 3 | 4 | 5 | 6 | 7 | 8 | 9 | 10 | 11 | 12 | 13 | 14 |
| --- | --- | --- | --- | --- | --- | --- | --- | --- | --- | --- | --- | --- | --- | --- |
| **Distal predictors** |  |  |  |  |  |  |  |  |  |  |  |  |  |  |
| 15. H | .11^***^ | -.13^***^ | .04 | -.01 | -.08 | .03 | .11^**^ | -.05 | -.06 | -.06 | -.08 | .02 | -.02 | -.14^***^ |
| 16. E | .37^***^ | -.05 | .02 | -.11^***^ | -.08 | .08^**^ | -.13^***^ | .07 | .13^***^ | -.05 | .06 | .13^***^ | .21^***^ | .03 |
| 17. X | -.04 | .15^***^ | .02 | .14^***^ | .00 | -.02 | .24^***^ | -.06 | .00 | .08^*^ | -.02 | -.05 | -.02 | -.17^***^ |
| 18. A | -.01 | .05 | -.01 | .05 | -.05 | .05 | .09^**^ | -.04 | .00 | -.01 | -.04 | .06 | -.01 | -.14^***^ |
| 19. C | .07 | -.17^***^ | .23^***^ | .10^***^ | -.02 | -.01 | .20^***^ | -.15^***^ | .05 | .03 | .01 | .10^**^ | -.02 | -.13^***^ |
| 20. O | .04 | -.17^***^ | .32^***^ | .14^***^ | -.05 | .00 | .15^***^ | -.11^***^ | .18^***^ | .15^***^ | .22^***^ | .12^***^ | -.02 | -.03 |
| 21. D | .03 | .11^**^ | -.16^***^ | -.14^***^ | .01 | -.06 | -.29^***^ | .16^***^ | .11^***^ | .02 | .11^**^ | .00 | .16^***^ | .19^***^ |
| 22. REI-R | -.05 | -.20^***^ | .34^***^ | .15^***^ | .02 | -.02 | .18^***^ | -.12^***^ | .06 | .09^**^ | .06 | .07 | -.06 | .00 |
| 23. REI-E | .12^***^ | -.25^***^ | .09^**^ | .09^**^ | -.06 | -.03 | .18^***^ | -.14^***^ | .19^***^ | .16^***^ | .18^***^ | .05 | .11^**^ | .03 |
| 24. AOT | .06 | -.23^***^ | .24^***^ | .12^***^ | -.12^***^ | .05 | .19^***^ | -.16^***^ | .05 | .04 | .08 | .10^**^ | -.08^**^ | -.05 |
| 25. CRT | -.15^***^ | -.06 | .13^***^ | .16^***^ | .04 | .07 | .10^**^ | -.02 | -.07 | .01 | -.05 | -.03 | -.10^**^ | -.01 |
| **Proximal predictors** |  |  |  |  |  |  |  |  |  |  |  |  |  |  |
| 26. Apophenia | .04 | .01 | .03 | .03 | .05 | .00 | .03 | -.04 | .05 | .00 | .03 | .06 | .04 | .06 |
| 27. CMQ | .04 | -.06 | .05 | -.08 | .08 | -.04 | .03 | .01 | .08 | .01 | .00 | .13^***^ | .07 | .08 |
| 28. MCT | .05 | .09^**^ | -.05 | -.15^***^ | .06 | -.08^**^ | -.09^**^ | .11^**^ | .17^***^ | .08^**^ | .05 | .11^***^ | .18^***^ | .09^**^ |
| 29. MHB | .04 | .24^***^ | -.12^***^ | -.10^**^ | -.01 | -.03 | -.11^***^ | .14^***^ | .18^***^ | .11^**^ | .06 | .06 | .21^***^ | .00 |
| 30. Superstition | .10^**^ | .12^***^ | -.11^**^ | -.06 | .01 | .01 | -.11^**^ | .07 | .06 | -.07 | -.02 | .00 | .22^***^ | .00 |
| 31. ESB | .16^***^ | .05 | .00 | -.05 | -.01 | -.04 | -.04 | .04 | .28^***^ | .16^***^ | .16^***^ | .14^***^ | .28^***^ | .04 |
| 32. Doublethink | -.06 | .10^**^ | -.12^***^ | -.12^***^ | .10^**^ | -.04 | -.06 | .09^**^ | -.12^***^ | -.09^*^ | -.20^***^ | -.02 | -.01 | -.01 |
| 33. GABS | .10^**^ | .01 | -.04 | -.12^***^ | .03 | .01 | -.13^***^ | .10^**^ | .02 | -.05 | -.03 | .04 | .08 | .08 |

*Note*.  ^***^ *p* < .001, ^**^ *p* < .01, ^*^ *p* < .05

*Correlations between variables (continued)*

|  | 1 | 2 | 3 | 4 | 5 | 6 | 7 | 8 | 9 | 10 | 11 | 12 | 13 | 14 |
| --- | --- | --- | --- | --- | --- | --- | --- | --- | --- | --- | --- | --- | --- | --- |
| **Social beliefs** |  |  |  |  |  |  |  |  |  |  |  |  |  |  |
| 34. Political orientation | .03 | -.03 | -.08^**^ | -.02 | .00 | .02 | -.02 | .02 | .09^**^ | .04 | -.03 | .06 | .17^***^ | .05 |
| 35. Religiousness | .09^**^ | .04 | -.09^**^ | -.01 | -.04 | .06 | -.01 | .06 | .16^***^ | .00 | -.02 | .04 | .39^***^ | -.04 |
| 36. Spirituality | .07 | -.03 | -.01 | .05 | -.10^**^ | -.04 | -.03 | -.05 | .23^***^ | .10^**^ | .25^***^ | .01 | .22^***^ | .05 |
| **Cognitive biases** |  |  |  |  |  |  |  |  |  |  |  |  |  |  |
| 37. Overconfidence | .10^**^ | .05 | -.09^**^ | -.13^***^ | -.01 | -.06 | -.05 | .02 | .10^**^ | .03 | .05 | .03 | .13^***^ | .01 |
| 38. Illusory corr. | .01 | .08^**^ | -.15^***^ | -.09^**^ | .04 | .06 | -.05 | .03 | -.04 | -.04 | -.03 | -.05 | .01 | -.11^***^ |
| 39. Naturalness | .05 | -.04 | .01 | -.02 | .04 | .06 | .04 | -.01 | .04 | .00 | -.04 | .06 | .09^**^ | -.01 |
| 40. Omission | -.02 | .07 | -.01 | -.02 | -.02 | .00 | -.04 | .06 | .07 | .06 | .04 | .02 | .07 | .03 |
| 41. Belief | .04 | .01 | -.18^***^ | -.08 | -.06 | -.04 | .06 | .02 | .02 | .02 | -.06 | .06 | .04 | -.07 |
| 42. Commitment | .00 | .07 | -.05 | .02 | .12^***^ | -.04 | .01 | .03 | -.01 | -.02 | -.03 | .04 | -.02 | .09^**^ |
| **Healthcare-related beliefs and experiences** |  |  |  |  |  |  |  |  |  |  |  |  |  |  |
| 43. Distrust in  medical system | .00 | -.12^***^ | .04 | -.10^**^ | .06 | -.05 | -.04 | .04 | .08^**^ | .02 | .07 | .09^**^ | .04 | .17^***^ |
| 44. Trust in  medical staff | -.03 | .18^***^ | -.07 | .12^***^ | -.02 | .06 | .10^**^ | .02 | -.10^**^ | -.08 | -.13^***^ | -.02 | -.04 | -.21^***^ |
| 45. Trust in science | -.09^**^ | .02 | .10^**^ | .12^***^ | .06 | .04 | .14^***^ | -.06 | -.12^***^ | -.07 | -.10^**^ | .01 | -.13^***^ | -.11^**^ |
| 46. Negative experiences  with medical system | .11^**^ | -.13^***^ | .02 | -.15^***^ | -.02 | -.01 | -.19^***^ | .09^**^ | .24^***^ | .18^***^ | .22^***^ | .08^**^ | .13^***^ | .28^***^ |

*Note*.  ^***^ *p* < .001, ^**^ *p* < .01, ^*^ *p* < .05

*Correlations between variables (continued)*

|  | 15 | 16 | 17 | 18 | 19 | 20 | 21 | 22 | 23 | 24 | 25 | 26 | 27 | 28 | 29 | 30 | 31 | 32 | 33 |
| --- | --- | --- | --- | --- | --- | --- | --- | --- | --- | --- | --- | --- | --- | --- | --- | --- | --- | --- | --- |
| **Distal predictors** |  |  |  |  |  |  |  |  |  |  |  |  |  |  |  |  |  |  |  |
| 15. H |  |  |  |  |  |  |  |  |  |  |  |  |  |  |  |  |  |  |  |
| 16. E | .08^**^ |  |  |  |  |  |  |  |  |  |  |  |  |  |  |  |  |  |  |
| 17. X | .07^*^ | -.16^***^ |  |  |  |  |  |  |  |  |  |  |  |  |  |  |  |  |  |
| 18. A | .26^***^ | -.07^*^ | .14^***^ |  |  |  |  |  |  |  |  |  |  |  |  |  |  |  |  |
| 19. C | .31^***^ | .04 | .21^***^ | .10^**^ |  |  |  |  |  |  |  |  |  |  |  |  |  |  |  |
| 20. O | .14^***^ | .00 | .14^***^ | .11^***^ | .23^***^ |  |  |  |  |  |  |  |  |  |  |  |  |  |  |
| 21. D | -.34^***^ | .13^***^ | -.37^***^ | -.19^***^ | -.39^***^ | -.10^**^ |  |  |  |  |  |  |  |  |  |  |  |  |  |
| 22. REI-R | .17^***^ | -.11^**^ | .22^***^ | .02 | .33^***^ | .42^***^ | -.20^***^ |  |  |  |  |  |  |  |  |  |  |  |  |
| 23. REI-E | .12^***^ | .02 | .13^***^ | -.04 | .15^***^ | .26^***^ | -.04 | .24^***^ |  |  |  |  |  |  |  |  |  |  |  |
| 24. AOT | .24^***^ | .03 | .02 | .15^***^ | .24^***^ | .33^***^ | -.32^***^ | .27^***^ | .10^**^ |  |  |  |  |  |  |  |  |  |  |
| 25. CRT | .03 | -.09^**^ | .03 | -.02 | .09^**^ | .12^***^ | -.16^***^ | .28^***^ | .00 | .23^***^ |  |  |  |  |  |  |  |  |  |
| **Proximal predictors** |  |  |  |  |  |  |  |  |  |  |  |  |  |  |  |  |  |  |  |
| 26. Apophenia | .02 | -.02 | .08^*^ | -.01 | .05 | .09^**^ | .02 | .06 | .07^*^ | -.06^*^ | -.07^*^ |  |  |  |  |  |  |  |  |
| 27. CMQ | .04 | .09^**^ | .02 | -.06^*^ | .12^***^ | .01 | .07^*^ | .09^**^ | .16^***^ | -.04 | -.01 | .05 |  |  |  |  |  |  |  |
| 28. MCT | -.05 | .09^**^ | .03 | -.04 | -.05 | -.11^***^ | .24^***^ | -.07^*^ | .13^***^ | -.29^***^ | -.15^***^ | .01 | .54^***^ |  |  |  |  |  |  |
| 29. MHB | -.08^**^ | .17^***^ | .11^***^ | .08^*^ | -.11^***^ | -.07^*^ | .31^***^ | -.10^**^ | .08^*^ | -.27^***^ | -.20^***^ | .01 | .30^***^ | .56^***^ |  |  |  |  |  |
| 30. Superstition | -.18^***^ | .22^***^ | -.01 | -.08^*^ | -.15^***^ | -.21^***^ | .32^***^ | -.16^***^ | -.03 | -.31^***^ | -.19^***^ | .03 | .10^**^ | .25^***^ | .37^***^ |  |  |  |  |
| 31. ESB | -.01 | .15^***^ | .01 | .00 | .07^*^ | .05 | .20^***^ | .00 | .22^***^ | -.05 | -.08^*^ | .03 | .30^***^ | .39^***^ | .46^***^ | .25^***^ |  |  |  |
| 32. Doublethink | -.06 | .08^**^ | .02 | -.01 | -.04 | -.16^***^ | .15^***^ | -.08^*^ | -.03 | -.30^***^ | -.15^***^ | .04 | .30^***^ | .31^***^ | .34^***^ | .28^***^ | .07^*^ |  |  |
| 33. GABS | -.26^***^ | .35^***^ | -.10^**^ | -.19^***^ | -.11^**^ | -.12^***^ | .30^***^ | -.08^*^ | .02 | -.19^***^ | -.12^***^ | .00 | .17^***^ | .19^***^ | .29^***^ | .35^***^ | .10^**^ | .31^***^ |  |

*Note*.  ^***^ *p* < .001, ^**^ *p* < .01, ^*^ *p* < .05

*Correlations between variables (continued)*

|  | 15 | 16 | 17 | 18 | 19 | 20 | 21 | 22 | 23 | 24 | 25 | 26 | 27 | 28 | 29 | 30 | 31 | 32 | 33 |
| --- | --- | --- | --- | --- | --- | --- | --- | --- | --- | --- | --- | --- | --- | --- | --- | --- | --- | --- | --- |
| **Social beliefs** |  |  |  |  |  |  |  |  |  |  |  |  |  |  |  |  |  |  |  |
| 34. Political orientation | .00 | .01 | .01 | .00 | -.04 | -.12^***^ | .08^**^ | -.05 | .04 | -.20^***^ | -.14^***^ | .03 | .08^**^ | .24^***^ | .18^***^ | .21^***^ | .13^***^ | .15^***^ | .10^**^ |
| 35. Religiousness | -.01 | .15^***^ | .14^***^ | .03 | .00 | -.08^*^ | .07^*^ | -.09^**^ | .04 | -.21^***^ | -.08^**^ | -.03 | .06 | .24^***^ | .22^***^ | .23^***^ | .27^***^ | .08^*^ | .07^*^ |
| 36. Spirituality | -.16^***^ | .08^**^ | -.02 | -.01 | -.08^**^ | .08^*^ | .18^***^ | -.04 | .10^**^ | -.03 | -.01 | .00 | -.03 | .06^*^ | .17^***^ | .16^***^ | .26^***^ | -.07^*^ | .06 |
| **Cognitive biases** |  |  |  |  |  |  |  |  |  |  |  |  |  |  |  |  |  |  |  |
| 37. Overconfidence | -.02 | .05 | .03 | .01 | -.07^*^ | -.08^*^ | .14^***^ | -.18^***^ | .03 | -.23^***^ | -.88^***^ | .04 | .05 | .19^***^ | .23^***^ | .19^***^ | .08^*^ | .18^***^ | .11^***^ |
| 38. Illusory corr. | .00 | .00 | -.01 | .07^*^ | -.05 | -.10^**^ | .06 | -.12^***^ | -.03 | -.12^***^ | -.15^***^ | .03 | -.06 | -.02 | .09^**^ | .08^*^ | .05 | .10^**^ | -.02 |
| 39. Naturalness | .07^*^ | .11^**^ | -.01 | -.05 | .03 | -.06 | .00 | .01 | .04 | -.03 | -.01 | -.04 | .18^***^ | .24^***^ | .19^***^ | .14^***^ | .17^***^ | .13^***^ | .12^***^ |
| 40. Omission | .03 | -.02 | -.01 | .04 | -.07^*^ | -.05 | .04 | -.04 | .07^*^ | -.08^*^ | -.08^*^ | -.01 | .05 | .16^***^ | .11^**^ | .06 | .09^**^ | .02 | .10^**^ |
| 41. Belief | .05 | -.02 | .06 | -.01 | -.01 | -.10^**^ | .05 | -.06 | .05 | -.17^***^ | -.21^***^ | .01 | .13^***^ | .21^***^ | .14^***^ | .13^***^ | .06 | .22^***^ | .02 |
| 42. Commitment | .02 | -.03 | .09^**^ | .01 | .02 | .00 | .07^*^ | -.05 | .05 | -.20^***^ | -.07^*^ | .07^*^ | .16^***^ | .20^***^ | .13^***^ | .05 | .11^**^ | .12^***^ | -.02 |
| **Healthcare-related beliefs and experiences** |  |  |  |  |  |  |  |  |  |  |  |  |  |  |  |  |  |  |  |
| 43. Mistrust in medical system | -.07^*^ | .07^*^ | -.07^*^ | -.13^***^ | -.02 | -.02 | .13^***^ | .06 | .10^**^ | -.05 | -.08^*^ | .01 | .29^***^ | .27^***^ | .13^***^ | .14^***^ | .12^***^ | .20^***^ | .19^***^ |
| 44. Trust in medical staff | .05 | -.01 | .18^***^ | .16^***^ | .00 | .04 | -.05 | .00 | -.06 | .02 | -.01 | .04 | -.14^***^ | -.20^***^ | .00 | .04 | -.05 | .00 | -.01 |
| 45. Trust in science | .01 | -.04 | .10^**^ | .10^**^ | .13^***^ | .17^***^ | -.17^***^ | .18^***^ | .01 | .21^***^ | .16^***^ | .08^*^ | -.10^**^ | -.33^***^ | -.17^***^ | -.11^**^ | -.14^***^ | .03 | .05 |
| 46. Negative experiences  with medical system | -.12^***^ | .12^***^ | -.11^***^ | -.11^**^ | -.10^**^ | .01 | .25^***^ | -.04 | .07^*^ | -.14^***^ | .10^**^ | .08^**^ | .06^*^ | .16^***^ | .06 | .05 | .06 | .03 | .14^***^ |

*Note*. ^***^ *p* < .001, ^**^ *p* < .01, ^*^ *p* < .05

*Correlations between variables (continued)*

|  | 34 | 35 | 36 | 37 | 38 | 39 | 40 | 41 | 42 | 43 | 44 | 45 | 46 |
| --- | --- | --- | --- | --- | --- | --- | --- | --- | --- | --- | --- | --- | --- |
| **Social beliefs** |  |  |  |  |  |  |  |  |  |  |  |  |  |
| 34. Political orientation |  |  |  |  |  |  |  |  |  |  |  |  |  |
| 35. Religiousness | .30^***^ |  |  |  |  |  |  |  |  |  |  |  |  |
| 36. Spirituality | .06 | .25^***^ |  |  |  |  |  |  |  |  |  |  |  |
| **Cognitive biases** |  |  |  |  |  |  |  |  |  |  |  |  |  |
| 37. Overconfidence | .15^***^ | .10^**^ | .01 |  |  |  |  |  |  |  |  |  |  |
| 38. Illusory corr. | .04 | .00 | -.01 | .13^***^ |  |  |  |  |  |  |  |  |  |
| 39. Naturalness bias | .10^**^ | .10^**^ | .03 | .02 | .05 |  |  |  |  |  |  |  |  |
| 40. Omission bias | .01 | .09^**^ | .07^*^ | .07^*^ | -.12^***^ | .06 |  |  |  |  |  |  |  |
| 41. Belief bias | .10^**^ | .07^*^ | -.12^***^ | .21^***^ | .07^*^ | .08^**^ | -.02 |  |  |  |  |  |  |
| 42. Commitment bias | .14^***^ | .10^**^ | -.04 | .11^**^ | .02 | .10^**^ | -.01 | .04 |  |  |  |  |  |
| **Healthcare-related beliefs and experiences** |  |  |  |  |  |  |  |  |  |  |  |  |  |
| 43. Mistrust in the medical system | .04 | .00 | -.01 | .10^**^ | -.09^**^ | .10^**^ | .05 | .08^*^ | .04 |  |  |  |  |
| 44. Trust in medical staff | -.01 | .03 | -.06 | .01 | .14^***^ | -.03 | -.01 | .01 | .01 | -.36^***^ |  |  |  |
| 45. Trust in science | -.11^**^ | -.12^***^ | -.08^*^ | -.12^***^ | .02 | -.07^*^ | -.05 | -.07^*^ | -.05 | -.09^**^ | .42^***^ |  |  |
| 46. Negative experiences with the medical system | .03 | .05 | .07^*^ | .09^**^ | -.10^**^ | .01 | .06 | -.02 | .02 | .31^***^ | -.42^***^ | -.24^***^ |  |

*Note*.  ^***^ *p* < .001, ^**^ *p* < .01, ^*^ *p* < .05
