# Supplementary figures and images for: Lifetime prevalence of questionable health behaviors and their psychological roots: A preregistered nationally representative survey
Source: PLoS One. 2024 Nov 6;19(11):e0313173. doi: 10.1371/journal.pone.0313173 (PMC11540216; doi:10.1371/journal.pone.0313173)

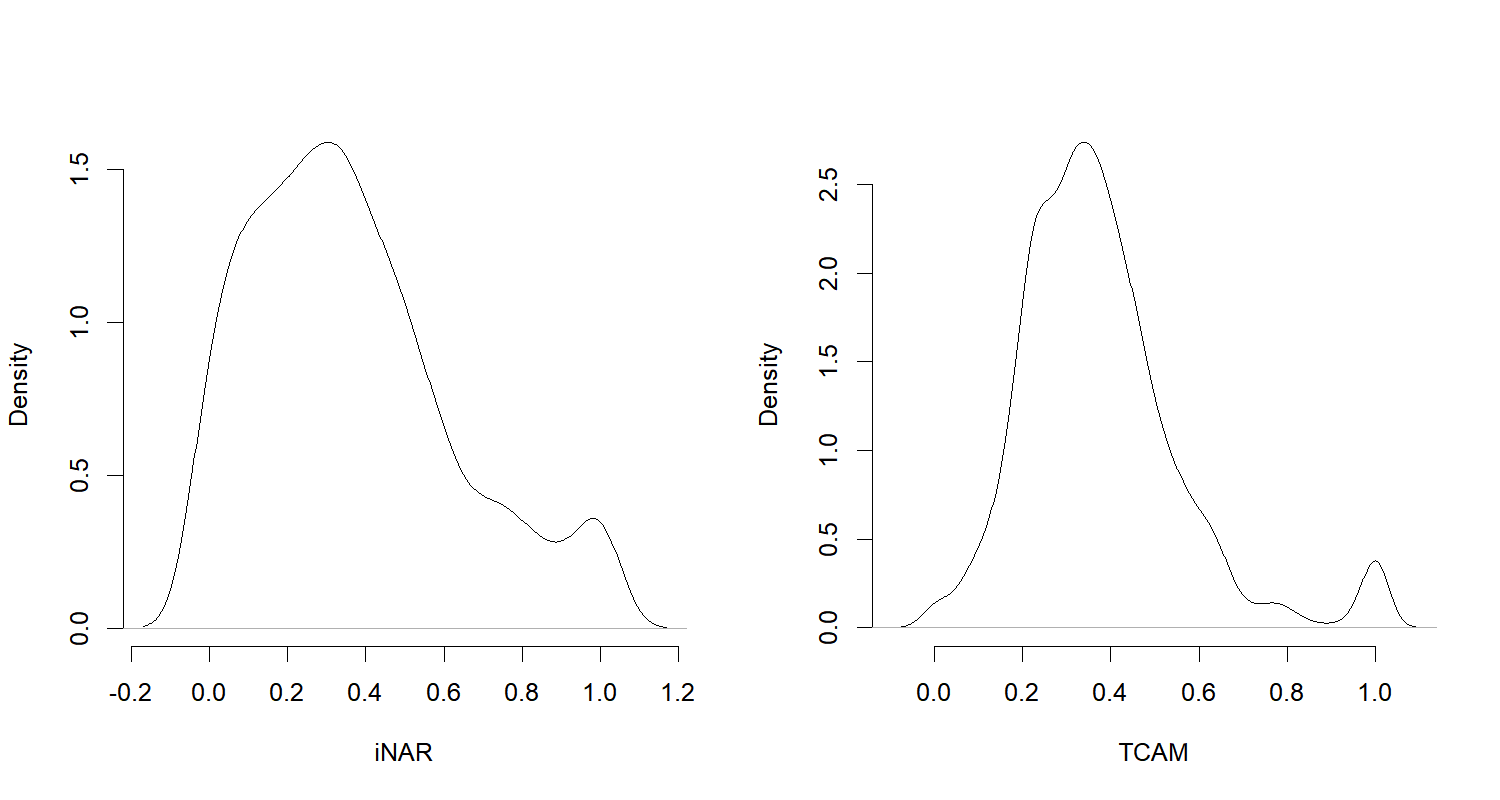

Supplement: S1 Fig — (TIF) [file pone.0313173.s005.tif]

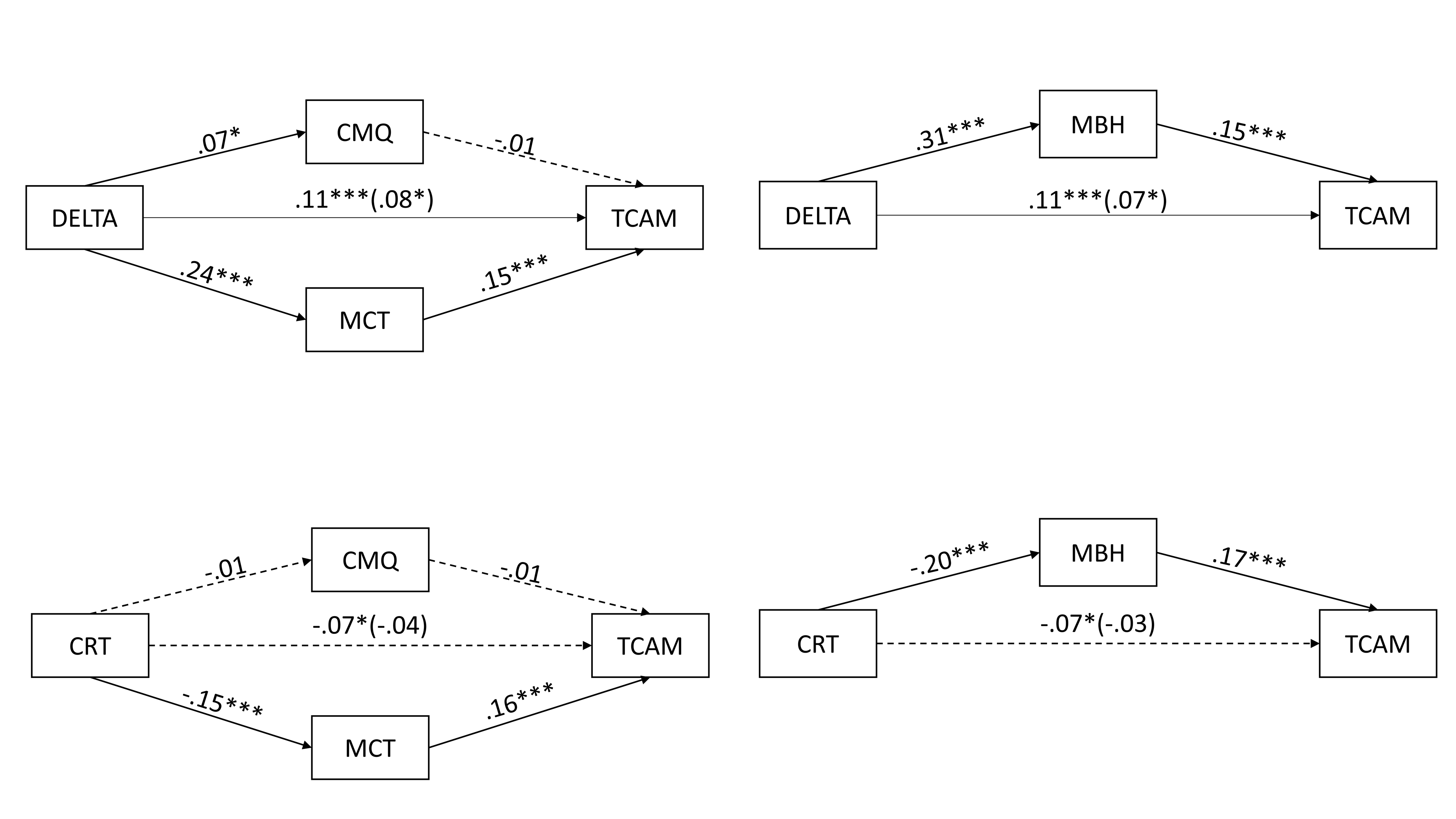

Supplement: S2 Fig — Disintegration and cognitive reflectivity as predictors, magical beliefs about health, general conspiracy mentality and medical conspiracy beliefs as mediators. CRT—Cognitive reflection; DELTA—Disintegration; MBH—Magical beliefs about health; CMQ—General conspiracy mentality; MCT—Medical conspiracy beliefs; TCAM—Use of traditional, complementary and alternative medicine. (TIF) [file pone.0313173.s006.tif]
